# Invertebrate Decline Has Minimal Effects on Oak‐Associated Microbiomes

**DOI:** 10.1111/1462-2920.70051

**Published:** 2025-02-12

**Authors:** Cynthia Albracht, François Buscot, Nico Eisenhauer, Alban Gebler, Sylvie Herrmann, Anja Schmidt, Mika Tarkka, Kezia Goldmann

**Affiliations:** ^1^ Institute for Biosafety in Plant Biotechnology Julius Kühn‐Institut Quedlinburg Germany; ^2^ Department of Soil Ecology Helmholtz Centre for Environmental Research – UFZ Halle Halle Germany; ^3^ German Centre for Integrative Biodiversity Research (iDiv) Halle‐Jena‐Leipzig Leipzig Germany; ^4^ Institute of Biology, Leipzig University Leipzig Germany; ^5^ Department of Soil System Science Helmholtz Centre for Environmental Research – UFZ Halle Halle Germany; ^6^ Department of Conservation & Social‐Ecological Systems Helmholtz Centre for Environmental Research – UFZ Leipzig Leipzig Germany

**Keywords:** bacteria, ecosystem functioning, fungi, iDiv Ecotron, insect decline, plant microbiome, *Quercus robur*

## Abstract

Recently, biomass of invertebrates has declined substantially at many locations with the implications of this biodiversity loss for ecosystems yet unknown. Through multitrophic interactions, plant‐ and soil‐associated microbiomes might be altered, causing a cascade of changes on diverse ecosystem processes. We simulated aboveground invertebrate decline in grassland ecosystems with two levels of invertebrate biomass (36% and 100% of current ambient conditions), plus a control with no invertebrates present. Each standardised grassland mesocosm additionally contained one clonal *Quercus robur* L. sapling to investigate the extent of invertebrate decline effects exceeding grasslands. We investigated oak biomass partitioning and mycorrhiza formation, oak leaf transcriptome and microbiome composition of leaves, roots and rhizosphere. While invertebrate decline did not significantly affect oak performance and herbivory‐related gene expression, fungal communities presented an increase of saprotrophs and pathogens, especially in leaves. Among leaf‐inhabiting bacteria, Proteobacteria and Actinobacteria increased under invertebrate decline. The belowground microbiome was only little affected. But, invertebrate decline came along with a reduced influence on predators leading to an elevated aphids infestation that proofed able to alter microbiota. Our findings establish a strong difference between above‐ and belowground, with the impacts of invertebrate decline being more pronounced in the leaf microbiome.

## Graphical abstract

Reduced invertebrate biomass impacts oak‐associated microbial communities: oak compartment specific bacterial and fungal communities showed divergent reactions to insect decline within an experimental set‐up in an ecotron facility, called ‘Insect Armageddon’.

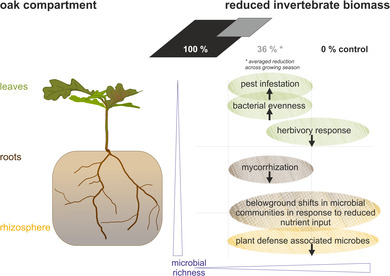

## 1 Introduction

Invertebrates are key players within ecosystems, being ubiquitous and providing a vast amount of services and functions: They provide beneficial services like pollination, pest control, nutrient cycling and decomposition (Hallmann et al. 2017; Losey and Vaughan 2006; Yang and Gratton 2014; Weisser and Siemann 2008; Jankielsohn 2018; Elizalde et al. 2020; Eisenhauer, Bonn, and Guerra 2019; Eisenhauer and Hines 2021; Scudder 2017), but can also have negative impacts as, for example, pests or invasive species (Elizalde et al. 2020). Land‐use changes, habitat destruction and climate disruption have led to a decrease of invertebrates in the last decades (Dirzo et al. 2014; Seibold et al. 2019). This arthropod decline is vast and noticeable across biomes; in Germany for example, biomass of arthropods declined by 67% in grasslands and by 41% in forest within 10 years (Seibold et al. 2019). Such decline affects multiple trophic levels and recent studies analysed, for instance, what are the consequences of invertebrate biomass loss on the abundance of pollination‐dependent plants (Biesmeijer et al. 2006; Gabriel and Tscharntke 2007), and eventually a drop of crop yields (Losey and Vaughan 2006). But, the far‐reaching and long‐term consequences of invertebrate decline on whole ecosystems and trophic interactions, while increasingly studied, are not yet fully understood.

Another group of understudied key drivers of ecosystem stability, are microorganisms (Yang et al. 2018). Microbes provide nutrients that enhance plant growth, can increase the competitive abilities of their associated plants and support defence mechanisms (Sharifi, Lee, and Ryu 2018; Sharifi and Ryu 2017). Being present in both plant and invertebrates, microbes mediate beneficial and detrimental multi‐trophic interactions (Shikano et al. 2017; Schädler et al. 2004). Therefore, when looking at impacts of invertebrate decline on ecosystems, plant‐ and invertebrate‐associated microbiomes need to be taken into consideration.

A common tripartite interaction of plants, invertebrates and microbes is herbivory. Invertebrates feeding on plants may alter the performance, structure and composition of plant communities (Schädler et al. 2004; Crawley 2009) as herbivory induces pathways of volatile production and defence mechanisms (De Vos et al. 2005). Moreover, invertebrate herbivory enhances and alters leaf microbial colonisation, and the rhizosphere microbiome is differentially shaped by shoot or root herbivory (Humphrey and Whiteman 2020; Friman et al. 2021). On the other hand, microbes alter the behaviour of herbivores by influencing the plants' defences mechanisms, and, for example, volatile profiles (Shikano et al. 2017). Through plant–soil feedbacks, beneficial microbes are enriched in relation to the type of herbivory and the identity of herbivores (Friman et al. 2021; Malacrinò et al. 2021). Herbivores drive above‐ to belowground nutrient fluxes in diverse ecosystems (Schowalter et al. 2011; Kaukonen et al. 2013; Hollinger 1986; Fonte and Schowalter 2005; Bacht et al. 2019; Lovett and Ruesink 1995), which impacts the quantity and quality of microbial‐driven decomposition and plant nutrient uptake, in some cases increasing plant growth and resistance (Yang and Gratton 2014; Belovsky and Slade 2000; Bardgett 2005). Thereby, these kinds of aboveground plant‐invertebrate interactions are regulated by a network of signalling pathways, which overlap with those of plant‐microbe interactions belowground (Yang and Gratton 2014; Schenk et al. 2008). Such belowground plant‐microbe interactions are mediated via root exudates (Sharifi and Ryu 2017; Bais, Broeckling, and Vivanco 2008; Bais et al. 2006; Broeckling et al. 2008; Narula, Kothe, and Behl 2012), and the complexity of these relationships is most apparent in the rhizosphere (Badri et al. 2009; Buée et al. 2009; Marschner 2007; Sweeney et al. 2021). Assembling a beneficial microbiome can enhance plant growth, but also influences nutritional and phytochemical compositions of plants (Sharifi and Ryu 2017; Fox et al. 2016; Kloppholz, Kuhn, and Requena 2011; Recourt et al. 1992). Subsequent changes in the structure of plant communities can affect interaction with invertebrates (Shikano et al. 2017; Kempel et al. 2013; Koricheva, Gange, and Jones 2009), and vice versa, invertebrate defaunation can impact plant community structures (Sharifi and Ryu 2017; Souza, Zelikova, and Sanders 2016).

Evidently, plants are essential for linking below‐ and aboveground processes (Wardle et al. 2004; Wardle 2002; Wurst 2013). The hotspots of plant‐invertebrate‐microbe interactions are leaves, roots and the rhizosphere (Blagodatskaya and Kuzyakov 2013). To better understand this linkage in the face of invertebrate declines, we exposed clonal saplings of pedunculated oak ( *Quercus robur* L.) to three levels of aboveground invertebrate abundance. *Q. robur* L. is as widely distributed across Europe (Iverson and Prasad 2001; Lévy, Becker, and Duhamel 1992) and attracts large communities of invertebrates (Brändle and Brandl 2001). We investigate the indirect effects of invertebrate decline both above‐ and belowground by analysing oak‐associated microbiome of roots, rhizosphere and leaves of these clonal oaks. We expect the decline of aboveground invertebrate biomass to (i) cause the strongest shifts in leaf microbial communities and a reduced expression of herbivory‐related plant genes under invertebrate loss conditions as leaves are at the direct interface of invertebrate loss via herbivory. We further assume a loss of aboveground invertebrate biomass (ii) modifies the community structures of oak‐associated bacteria and fungi in rhizosphere soil and roots. Finally, we address whether (iii) invertebrate loss affects overall tree performance through changes in nutrient availability and microbe interaction.

## 2 Material and Methods

### 2.1 Experimental Design

This study was part of the Insect Armageddon experiment investigating consequences of insect decline above‐ and belowground artificial grassland communities and clonal oaks. We manipulated aboveground invertebrate biomass in 24 identical experimental chambers (EcoUnits, Figure S1a) of the iDiv Ecotron (Eisenhauer and Türke 2018). This experimental facility located in the research station of the Helmholtz Centre for Environmental Research (UFZ) in Bad Lauchstädt, Germany (51° 22′ 60 N, 11° 50′ 60 E, 118 m a.s.l.), allows continuous monitoring of mesocosms under controlled environmental conditions. The EcoUnits were set to a standardised 12:8 h day:night cycle with an average temperature of 24°C during daytime and 19°C at night. Irrigation was set at 6 L of deionised water per day. Each EcoUnit mesocosm with an internal area of ~1.54 m (Losey and Vaughan 2006) and a depth of 0.8 m, was filled with 1.23 m^3^ of a sieved (15 mm) mixture of topsoil (80%) and sand (20%), inoculated with soil from an adjacent hay meadow. In April 2018, a standardised plant community was grown from seeds in each of the EcoUnits (Figure S1b), comprising three grass species ( *Arrhenatherum elatius* (L.), P. Beauv. ex J. Presl and C. Presl, *Phleum pratense* L. and *Dactylis glomerata* L.), eight predominantly invertebrate‐pollinated herbs ( *Centaurea jacea* L. s. l., *Lotus corniculatus* L., *Medicago lupulina* L., *Scorzoneroides autumnalis* (L.) Moench, *Trifolium pratense* L., *Achillea millefolium* L., *Knautia arvensis* (L.) Coult. and *Bellis perennis* L.), and one primarily anemochorous species ( *Plantago lanceolata* L.) representative for a tall oatgrass (*Arrhenatherion elatioris*) meadow and inspired by the same hay meadow soil inoculants were taken from. Further details on plant species selection and planting procedure of the plant community can be found in Ulrich et al. (2020). Each EcoUnit additionally contained one DF159 oak clone sapling ( *Q. robur* , Herrmann, Munch, and Buscot 1998, with a height of 30–45 cm when planted). The DF159 clones were produced by micropropagation and pre‐cultivated in the greenhouse (16:6 h day:night cycle, 25°C) in 1 L soil mixture (1:1 soil from an oak plot in Kreinitz, Germany and the plant substrate *Frühstorfer Erde*, further information on soil in Table S1). The oak saplings were pre‐inoculated with the mycorrhizal fungus *Piloderma croceum* and were transferred to the EcoUnits in tubes of 3 L volume after formation of dormant buds but before total leaf abscission. To enable the determination of direct and indirect top‐down impacts, the oak roots were kept in these tubes as separated soil compartments throughout the experiment (Figure S1c).

To simulate invertebrate decline, we aimed for a three‐level treatment of invertebrate community biomass with high, mid and no invertebrate biomass. This was realised by collecting aboveground invertebrate species alive from an adjacent hay meadow (same as soil inoculants) with modified Malaise traps similar to Hallmann et al. (2017) and sweep‐net sampling following Seibold et al. (2019). Different quantities of caught invertebrates were realised through modification of the catching area and time span of catching (24 h with 36% catching area traps, 2 × 24 h with 100% catching area traps) and by number of net sweeps (2× and 8× each time, respectively). The standard sampling bottles of Malaise traps were replaced by invertebrate rearing cages (BugDorm, 30 × 30 × 30 cm) equipped with wadded‐up moistened paper towels to provide shelter and humidity, so the invertebrates stayed alive. No invertebrates were collected for the control treatment (0% invertebrate abundance).

The invertebrates were transferred to the EcoUnits immediately after the collection of 24 h/48 h duration, creating two levels of invertebrate biomass (IB) of high and mid invertebrate biomass, plus a control with no invertebrates added, with eight EcoUnit replicates for each of these treatments. To include a wide range of functionally important invertebrates and simulate seasonal turnover in their community composition, new invertebrates were collected at monthly intervals from May until September 2018 (timeline: Figure S2) and added to the EcoUnits while the previous communities were removed with a suction sampler (modified vacuum cleaner, Bosch Industriestaubsauer GAS 25). These monthly removed invertebrates were transferred to 70% ethanol for identification, weighing and counting. To assess the actual amount and reduction of invertebrates, biomass [g], abundance and richness of invertebrates of each addition/suction campaign were calculated, as described in Eisenhauer et al. (2023). Three samples were extrapolated to calculate invertebrate abundance, biomass and richness. To account for different sample collection methods, pairs of available Malaise samplings and one cup catch per Malaise for each treatment‐sampling round were combined creating 100 randomised datasets of pairings. From these randomised dataset, we calculated mean and SE, resulting in 100 mean values of richness, abundance and biomass to assess the invertebrate reduction throughout the experimental period.

### 2.2 Oak Traits and Harvest

We assessed shoot and leaf growth and growth stages of all oak clone saplings monthly, but here we focus on their traits at the time point of harvest (Figure S1d). At the end of the growing season in November 2018, the oaks were taken out as a whole, branches and stems were measured and shoot growth flushes counted. Leaves were counted, and a subsample of five leaves per shoot flush used to document leaf area and fresh weight. The root bale was freed of soil and the lateral roots separated from the primary roots for fresh weights. Overall mycorrhization was assessed by checking for the characteristic yellow hyphae mantle of *P. croceum* (Herrmann, Oelmuller, and Buscot 2004).

### 2.3 Oak‐Associated Microbiome

For the analysis of oak‐associated microbiomes, we collected samples of rhizosphere soil (RS), roots (RO) and leaves (LV) of the eight oak saplings replicates for each of the three treatments. Fine roots were cut from root bale and washed with deionised water to remove rhizosphere soil. The root wash water was filtered over a vacuum pump (polycarbonate filters with 0.2 μm pores) to collect the rhizosphere soil. The filters containing rhizosphere soil, the washed fine roots and 3–5 leaves per oak were bagged and frozen at ‐80°C. Additional 3–5 leaves were taken from the first growth flush of each oak sapling and directly frozen in liquid nitrogen, to analyse regulation of genes involved in herbivory defence. Additional soil samples were taken from each tube containing an oak sapling, tried and send to AGROLAB Agrarzentrum, Leinefelde‐Worbis, Germany for analyses of soil pH and N, C contents and soil type to be considered for further analyses.

### 2.4 Library Preparation and Amplicon Sequencing

In preparation of amplicon sequencing fungi and bacteria, DNA was extracted from fine roots, rhizosphere soil and leaves. We used different extraction kits for best yield: we used PowerSoil DNA Isolation Kit (Qiagen, Hilden, Germany) to extract DNA from one cut up filter full of rhizosphere soil; 80 mg of leaves ground in liquid nitrogen were processed with DNeasy Plant Mini Kit (Qiagen, Hilden, Germany). Roots were powdered in liquid nitrogen and 200 mg used to extract DNA with Quick‐DNA Fecal/Soil Microbe Miniprep Kit (Zymo Research, Freiburg, Germany), for all following manufacturers' instructions. DNA purity and quantity were measured with a NanoDrop spectrometer (Thermo Fisher Scientific, Waltham, MA, USA). For fungal communities, we amplified the target region ITS2 in triplicates using primers *P5‐5N‐ITS4* and *P5‐6N‐ITS4* together with *P7‐3N‐fITS7* and *P7‐4N‐fITS7* (Ihrmark et al. 2012; Gardes and Bruns 1993; Leonhardt et al. 2019), where P5/P7 are Illumina adapter sequences and *N* refers to the number of random nucleotides between adapter and target primer (Moll et al. 2021). For prokaryotes, we targeted the 16S rRNA gene for amplification with primers *P5‐8N‐515f* and *P5‐7N‐515f* together with *P7‐2N‐806r* and *P7‐1N‐806r* (Moll et al. 2021; Caporaso et al. 2011). The PCR mix for both targets was composed of 7.50 μL 2× KAPA HiFi HotStart Ready Mix (Roche Diagnostics, Mannheim, Germany), 0.30 μL of forward/reverse primers each, 5.40 μL nuclease free water and 1.50 μL template DNA. The thermo‐cycle conditions for fungi (ITS2) were 5 min at 95°C, then 30 cycles of 20 s at 98°C, 15 s at 56°C, and 15 s at 72°C with finally 5 min at 72°C, while prokaryotes (16S rRNA) were amplified with 3 min at 95°C, 25 cycles of 2 s at 98°C, 15 s at 55°C and 15 s at 72°C, finished with 5 min at 72°C. The PCR product triplicates were pooled and cleaned with AMPure XP magnetic beads (Beckman Coulter, Krefeld, Germany) before and after adding sequencing indices with Nextera XT index Kit (Illumina, Berlin, Germany). The purified PCR products were then quantified by a Quant‐IT PicoGreen dsDNA Assay Kit (Fisher Scientific, Schwerte, Germany) and combined to equimolar pools of ITS2 and 16S amplicons. These pools were quantified again with Qubit dsDNA HS Assay and Qubit 3 Fluorometer (Invitrogen by Fisher Scientific, Schwerte, Germany) and quality checked with High Sensitivity DNA Kit of an Agilent 2100 Bioanalyzer (Agilent Technologies, Frankfurt, Germany). The sequencing was performed at the Illumina MiSeq platform at the Department of Soil Ecology at Helmholtz Centre for Environmental Research—UFZ, Halle (Saale), Germany, as paired‐end sequencing of 2 × 300 bp.

### 2.5 Data Processing and Analyses

Raw sequence reads were processed with default settings of the dadasnake pipeline, version 0.11 (Weißbecker, Schnabel, and Heintz‐Buschart 2020) at the high‐performance computing cluster EVE (Helmholtz Centre for Environmental Research—UFZ/German Centre for Integrative Biodiversity Research (iDiv)). Filtered and trimmed sequences were assembled to clustered (97% sequence similarity) amplicon sequence variants (OTU) (Moll et al. 2024), and taxonomy was annotated with mothur and databases UNITE version 10.0 for fungi (Nilsson et al. 2018) and SILVA v132 for bacteria (Quast et al. 2012). Fungi were additionally checked with ITSx (Bengtsson‐Palme et al. 2013) to remove any false results and annotated with FungalTraits for their primary lifestyle (i.e., fungal guild) (Põlme et al. 2020).

All statistics were performed in R version 4.1.1 (R Core Team 2019). Reads were rarefied to lowest read number per sample to calculate OTU richness, and additionally Shannon diversity and Pielous' evenness to also assess shifts in the dominance of species. These and relative abundances were calculated with phyloseq (McMurdie and Holmes 2013). To analyse effects of invertebrate biomass and plant compartments on alpha diversities, linear models were calculated using the model: Alpha Div Index ~ Invertebrate Biomass (100%, 36%, and Control) × Oak Compartment (Roots, Rhizosphere Soil, Leaves). To show invertebrate biomass and plant compartment effects on community structures, Jensen‐Shannon dissimilarity (JSD) matrices were calculated with phyloseq (McMurdie and Holmes 2013). Communities were visualised as non‐metric multidimensional scaling (NMDS) based on Jensen‐Shannon divergence for fungi and euclidean distances for bacteria, according to data composition, using packages vegan (Oksanen et al. 2019) and ggplot2 (Wickham 2016). Shifts in microbial communities were statistically tested with a PERMANOVA on Jensen Shannon divergence in vegan (Oksanen et al. 2019) with the model distance matrix ~ Invertebrate Biomass × Oak Compartment and an ad‐hoc TukeyHSD test. Differential abundances were calculated with package ANCOMBC (Lin and Peddada 2020) to detect changes in fungal and bacterial abundances as affected by invertebrate biomass. The number of shared and individually present OTUs was visualised with VennDiagram (Chen and Boutros 2011).

Due to an infestation by aphids, that occurred throughout the experiment, the fungal and bacterial communities were additionally tested against aphid biomass. For this, JSD matrices as well as the alpha diversity indices were tested with a PERMANOVA against aphid biomass and plant compartment (Fungi/Bacteria ~ Aphids Biomass * Oak Compartment). Aphid biomass was measured together with the monthly addition/suction campaign of all invertebrates. Two EcoUnits had to be excluded from these analyses, due to missing invertebrate biomass data, leaving a total of 22 EcoUnits. All EcoUnits were equally treated with a standard insecticide (Karate Zeon, Raiffeisen) after the first grassland biomass harvest to resolve the infestation issue and the invertebrate treatments were re‐established afterwards.

### 2.6 Oak Leaf Transcriptomics

To analyse shifts in herbivory, oak leaf samples were ground in liquid nitrogen, and RNA was extracted from 30 mg using the RNeasy Plant Mini Kit (Qiagen, Hilden, Germany) with the additional RNAse‐free DNAse set (Qiagen, Hilden, Germany). The RNA extracts were verified with a formaldehyde gel electrophoresis (90 min at 50 V|2 μL denatured RNA + 2 μL loading buffer). Concentration of RNA extracts was determined with NanoDrop 8000 spectrometer (Fisher Scientific, Schwerte, Germany) to then dilute extracts to 8 ng/μL. Real time RT‐qPCR was performed with iTaq Universal One‐Step RT‐qPCR Kit with SYBR Green as fluorescent compound (Bio‐Rad Laboratories, Feldkirchen, Germany) with a reaction mix containing 7.50 μL iTaq universal SYBR Green reaction mix (2×), 0.30 μL iScript reverse transcriptase, 0.45 μL of forward/reverse primer each, 5.30 μL nuclease‐free water and 1.00 μL RNA (8 ng/μL). Five different primer pairs were chosen based on the findings of Bacht et al. (2019) to assess the effect of invertebrate abundance on herbivory‐related processes. Primers *Qr_13LOX3_F3*/*Qr_13LOX3_R3* (13‐lipoxygenase) and *Qr_AOC_F7*/*Qr_AOC_R7* (allene oxide cyclase) are related to the jasmonic acid biosynthesis, while primers *Qr‐Far4_F21*/*Qr‐Far4_R21* (alpha farnesene synthase) and *Qr_HPL4_F27*/*Qr_HPL4_R27* (hydroperoxide lyase) are connected to volatile production. Primers *Qr_CHI_F37/Qr_CHI_R37* for *chitinase* I were used as marker for a general herbivory‐induced response and 18S rRNA was used as standard. All primer sequences and IDs are listed in Table S2. The qRT‐PCR was run in an iQ5 Multicolor RealTime PCR Detection system (Bio‐Rad Laboratories, Feldkirchen, Germany) with the program: 10 min at 50°C, 5 min at 95°C, then 40 cycles of 10 s at 95°C, and 30 s at 60°C and finally 10 s at 60°C.

The raw data of qPCR Ct values were analysed using delta Ct analysis (Livak and Schmittgen 2001). The Ct values of the tested genes were related to Ct values of stably expressed 18S as a reference to whole RNA. Double delta Ct values (∆∆Ct) were calculated with the 0% invertebrate abundance as control and exponentiated to get fold changes. Statistical analyses were done in R with functions anova and TukeyHSD of the package vegan (Oksanen et al. 2019).

## 3 Results

### 3.1 Reduced Invertebrate Biomass

We established 24 grassland ecosystems in independent chambers (EcoUnits) at the iDiv Ecotron facility with a standardised plant community and three treatments of aboveground vegetation‐associated invertebrates. The usage of different sampling methods resulted in three treatment levels of 100%, 36% and 0% invertebrate biomass, simulating the invertebrate biomass decline across German grasslands as reported by Seibold et al. (2019). We obtained a mean biomass reduction of 64% across the whole season, with the highest biomass reduction in June at 82% (Table S7). Invertebrate abundance was reduced on average by 70% and richness by 44%. We also successfully simulated a seasonal turnover, with changing compositions of the invertebrate communities (Table S8–S11: Abundance and richness of taxonomic groups per month and treatment). Most abundant broad taxonomic group throughout the season were *Brachycera*, except in June when *Aphidina* dominated.

Though the establishment of the invertebrate biomass treatments was successful, we observed an aphids infestation within all EcoUnits in weeks 5 through 18 of the experiment. This aphid infestation was more intense at lower invertebrate biomass (0% and 36%) (Figure S3). While the aphids primarily targeted the grassland species and not the oaks, we tested for any indirect impacts this might have had on the oak microbiome and found aphid infestation to significantly affect α‐ and β‐diversities of the oak‐associated fungi, but only α‐diversity of bacterial communities (Table 1). This aphid infestation had no interactive effect with the compartments and resulted in tendentially higher microbial richness at higher aphid biomass. Soil pH and organic content of the soil did not shift under aphid infestation (Table S4). The invertebrate treatments were re‐established after insecticide application.

**TABLE 1:** Effect of aphid infestation on alpha diversity indices of fungi and bacteria tested with linear model and its effects on fungal and bacterial beta diversity tested with PERMANOVA; in bold significant *p* values (*p* ≤ 0.05).

		Aphid biomass (g)		Compartment		Aphid biomass * compartment	
		*F*	*p*	*F*	*p*	*F*	*p*
Fungi	OTU richness	6.04	**0.02**	71.67	**< 0.01**	0.63	0.54
	Shannon diversity	4.18	**0.04**	13.216	**< 0.01**	0.15	0.86
Bacteria	OTU richness	7.87	**< 0.01**	840.72	**< 0.01**	2.49	0.09
	Shannon diversity	1.88	0.17	643.14	**< 0.01**	1.34	0.27

		*R* ^2^	*p*	*R* ^2^	*p*	*R* ^2^	*p*
Fungi	β‐diversity	0.02	**0.04**	0.53	**< 0.01**	0.01	0.77
Bacteria	β‐diversity	< 0.01	0.18	0.84	**< 0.01**	< 0.01	0.37

Soil chemical properties of soil surrounding the saplings did also not differ much between the treatments, i and we did not find any effect of nvertebrate or aphid biomass on soil pH, organic substance, soil N or C:N ratio.

### 3.2 Oak Traits

Oak traits were monitored monthly to analyse impacts of declining invertebrate biomass (IB) on tree performance. At first, the oaks were overshadowed by the grassland plant community and only started to grow noticeably after mowing the grass at the end of August (data not shown). However, we did not find significant differences of oak growth between the treatments. Biomass partitioning (developmental stage) in the oak saplings was evaluated at harvest (Table S3) revealing primary roots with a tendency of higher mean fresh weight at 0% IB, while lateral roots had highest fresh weight at 100% IB. Mycorrhization rate of lateral roots was lowest at 36% IB and highest at 100% IB. Moreover, mean leaf fresh weight was lowest at 100%. However, none of the differences in oak performance above‐ and belowground were significant in response to insect biomass due to overall strong variations in growth between the oak saplings.

### 3.3 Oak Leaf Transcriptomics

**FIGURE 1:**
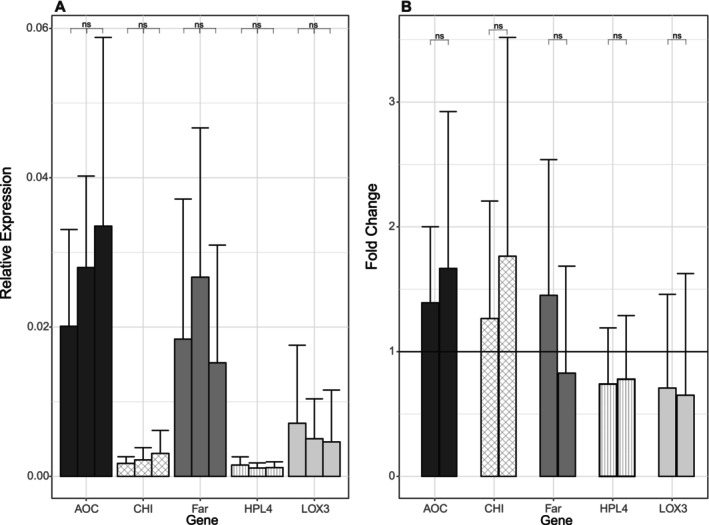
(a) Relative expression of herbivory‐related genes in leaves of oak tree clone DF159 under influence of 0% invertebrate (left bar), 36% invertebrate (mid) and 100% invertebrate biomass treatment (right bar) as normalised Ct‐value with indication of standard error. *AOC*—allene oxide cyclase; *LOX3*–13‐lipoxygenase; *HPL4*—hydroperoxide lyase; *Far*—alpha farnesene synthase; *CHI*—chitinase I. (b) Fold changes of gene expressions at 36% (left bar) and 100% IB (right) in comparison to 0% IB.

To assess the effect of IB on herbivory‐related responses, we performed real‐time RT‐qPCR on oak leaves. Expression of *AOC* and *CHI* increased with IB with fold changes of 1.4 and 1.3 at 36% and 1.7 and 1.8 at 100% IB, respectively, whereas expression of *LOX3* decreased with invertebrate biomass (Figure 1). *Far* had the highest expression at 36% IB with 1.4‐fold change, while *HPL4* had the lowest expression at this IB treatment with a 0.7‐fold change. However, the gene expression had high variations among the oak saplings and the invertebrate biomass did not have significant effect on gene expressions for any of the genes.

### 3.4 Microbial Alpha Diversity

We obtained a range of 55,589 to 97,985 fungal ITS reads per sample, which were assigned to 1501 OTUs. For bacteria, 8032 OTUs were assembled from a total of 6,488,442 reads ranging from 175 to 239,626 per sample. The reads were rarefied to the smallest read numbers per sample, which led to the removal of 60 fungal and 3884 bacterial OTUs plus two leaf samples of the bacteria dataset to due insufficient read numbers.

Among the fungi, 235 OTUs were only present at 0% IB, 287 fungal OTUs only at 36% IB and 212 fungal OTUs were only present at 100% IB (Figure S4). A total of 486 fungal OTUs were shared among all IB treatments. For bacteria, the majority of OTUs were shared between all IB treatments, while there were 764 specific bacterial OTUs at 0% IB treatments, 973 at 36% IB and 953 at 100% IB, respectively. Differentiating the oak compartments roots (RO), rhizosphere (RS) and leaves (LV), RS contained the highest number of specific OTU and had the most shared OTU with roots for both fungi and bacteria. Present in all three compartments were 274 fungal OTUs, and 280 bacterial OTUs.

**FIGURE 2:**
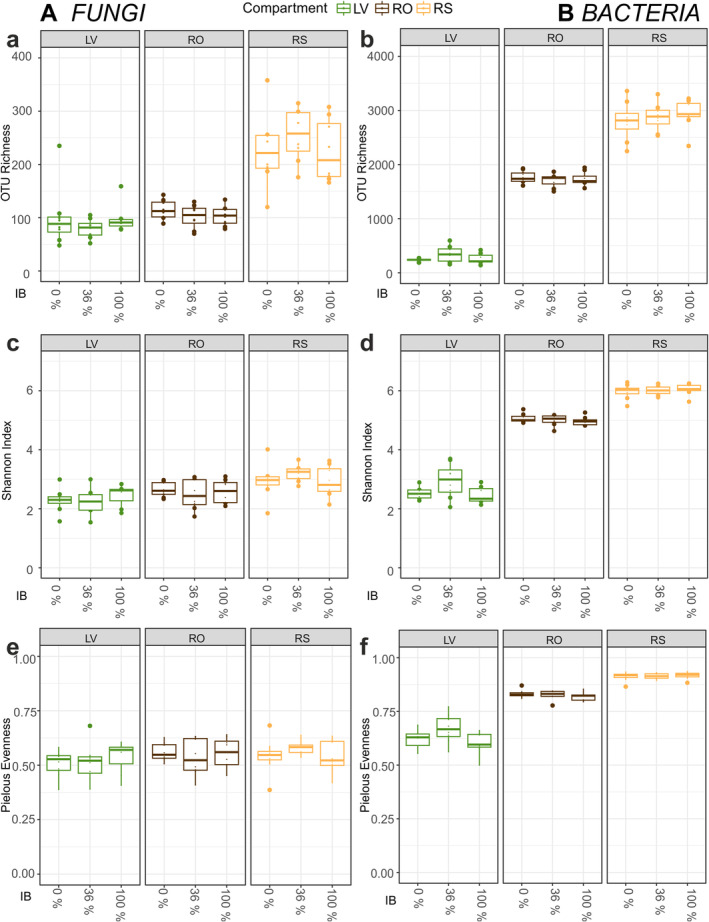
Alpha diversity indices of fungi (left) and bacteria (right) based of rarefied data: (a, b)—OTU richness, (c, d)—Shannon diversity, (e, f)—Pielous' Evenness as affected by IB (0%/36%/100%) and oak compartments (LV—leaves, RO—roots, RS—rhizosphere). Results of linear models for the relation of alpha indices and experimental IB treatments are indicated by *p* values (ns—non‐significant).

The microbial alpha diversity differed strongly between compartments, with the lowest OTU richness of both fungi and bacteria found in LV and highest OTU richness in RS (Figure 2). A linear model affirmed a strong significant effect of compartment on OTU richness (fungi: *F* = 80.88, *p* < 0.001| bacteria: *F* = 923.31, *p* < 0.001), as well as Shannon diversity (fungi: *F* = 15.64, *p* < 0.001|bacteria: *F* = 836.35, *p* < 0.001), but only on Pielou's evenness of bacteria (fungi: *F* = 1.83, *p* = 0.17|bacteria: *F* = 357.51, *p* < 0.001). The IB treatments, however, did overall not affect the alpha diversity indices of neither fungi nor bacteria. Looking at the oak compartments individually, IB had only a marginal effect on OTU evenness (*F* = 2.77, *p* = 0.09) and Shannon diversity (*F* = 2.9, *p* = 0.08, Table S5) of bacteria on LV.

### 3.5 Microbial Beta Diversity

**FIGURE 3:**
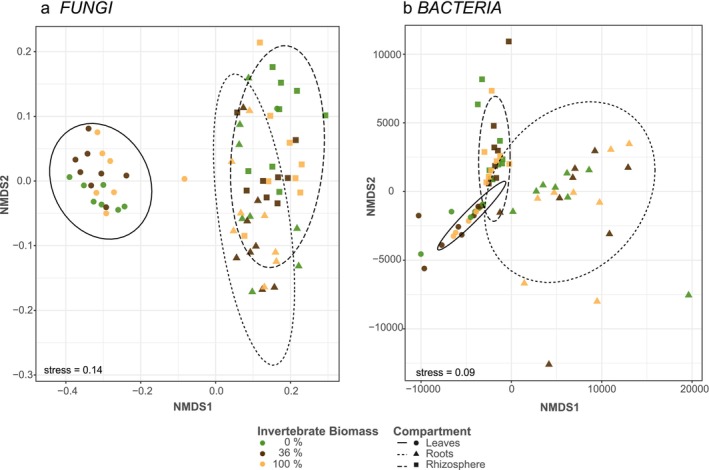
Oak microbiome plotted on NMDS for (a) fungal communities based on Jensen‐Shannon dissimilarity (stress = 0.14); and (b)—bacterial communities based on Euclidean distances (stress = 0.09). Plant compartments are displayed by shapes (leaves—circle, roots—triangle, rhizosphere—square) and invertebrate biomass treatments are differentiated by colours (0%—green, 36%—brown, 100%—yellow).

To assess microbial community structure, we calculated Jensen‐Shannon dissimilarities. Both fungal and bacterial communities clustered strongly by plant compartment (Figure 3), even though stress value for fungi was a little low at 0.14, and showed significant differences of fungal (*R* ^2^ = 0.54, *p* < 0.001) and bacterial (*R* ^2^ = 0.84, *p* < 0.001) communities in the three compartments. Reduced invertebrate biomass caused slight but significant shifts in fungal communities (*R* ^2^ = 0.03, *p* = 0.03), but had only marginal effects on bacterial communities (*R* ^2^ = 0.01, *p* = 0.08). We further could not find an interactive effect of invertebrate biomass and compartment on either fungi (*R* ^2^ = 0.01, *p* = 0.98) or bacteria (*R* ^2^ < 0.01, *p* = 0.37). None of these results was caused by differences in group dispersion.

### 3.6 Taxonomic Profiles of Oak‐Associated Microbiomes

**FIGURE 4:**
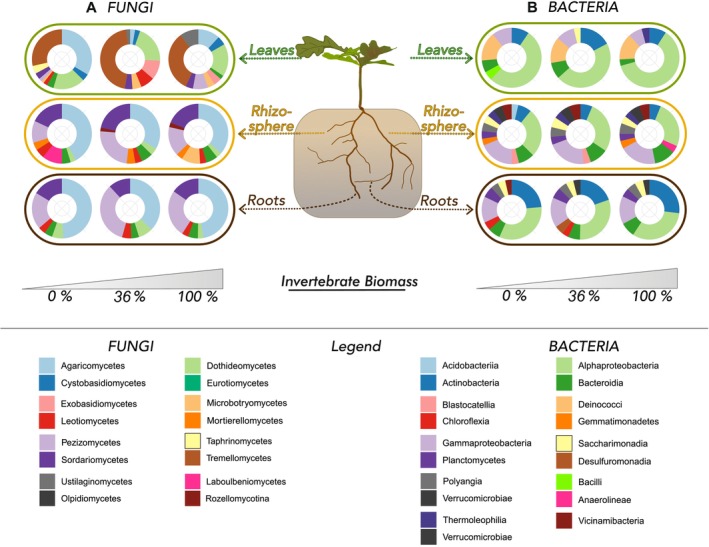
Relative abundances of (a) fungi and (b) bacteria aggregated at class level and filtered for > 2% relative abundance shown per invertebrate abundance (0%/36%/100%) and oak compartments (leaves, roots and rhizosphere).

The differences of relative abundances of fungi and bacteria aggregated at class level and filtered to > 2% relative abundance are shown in Figure 4. Fungal communities in RO and RS had similar taxonomic profiles dominated by *Agaricomycetes, Pezizomycetes and Sordariomycetes*, while the most abundant fungal class on leaves were *Tremellomycetes*. We found similar differences among the compartments for fungal guilds: leaf community were mainly comprised of mycoparasites, while RO and RS were dominated by ectomycorrhiza (Figure S5). For bacterial communities, *Alphaproteobacteria* were most abundant across compartments (Figure 4). RO and LV shared a high abundance of *Actinobacteria*, while RS bacterial communities contained a more diverse taxonomic profile, including *Blasticatellia* and *Gemmatimonadetes*, which were only abundant in the rhizosphere.

We tested differential abundances of fungal classes and bacterial phyla at 36% and 100% IB compared to the 0% IB and could only found few significantly differential abundant taxa. Among fungi on leaves, *Ustilagomycetes* were about 4× more abundant in presence of invertebrates at both 36% and 100% IB (Table S6). *Exobasidiomycetes* on leaves were 3× more abundant with invertebrates present and *Microbotryomycetes* only increased in abundance at 36% IB. Belowground, abundances of fungal classes were not significantly affected by IB, except for *Dothideomycetes* in roots being slightly less abundant at 100% IB.

For bacteria, as well, we did not see stringent results of relative abundances in response to invertebrate biomass. Among the bacterial leaf communities, *Bdellovibrionota* were 1.5× more abundant at 36% IB. We found candidate phylum FCPU426 to be 1.5× more abundant at 100% IB in both roots and rhizosphere. In roots, candidate phylum WPS‐2 (*Eremiobacterota*) was less abundant only at 36% IB. *Acidobacteria* were minimally less abundant at 100% IB.

## 4 Discussion

Our experiment highlighted the consequences of invertebrate biomass decline on ecosystems, providing the opportunity to study top‐down effects on above‐ and belowground. The usage of the iDiv Ecotron facility allowed for controlled environmental conditions further controlled by a standardised grassland community and targeted manipulation of aboveground invertebrate biomass. Within the iDiv Ecotron, we successfully simulated an invertebrate decline averaging at 36% invertebrate biomass across the experimental period for the reduced invertebrate treatment versus 100% and a control of no invertebrate biomass. Though this reduction was not a consistent level, the monthly addition/suction campaigns replicated seasonal turnovers, making the manipulation more realistic. For the experiment Insect Armageddon, it was already shown that the loss of invertebrate biomass decreases ecosystem multifunctionality and coupling, posing a threat towards the integrity of grasslands (Eisenhauer and Hines 2021). It was further found that declining invertebrate biomass leads to increased grassland biomass, while belowground decomposition and aboveground pest control are reduced (Eisenhauer and Hines 2021). This reduced top‐down control presented itself in form of an increased infestation by aphids at low total invertebrate biomass, which is in line with earlier studies finding a general invertebrate decline to not affect all taxa equally, as some benefitting insect species actually increase (Dirzo et al. 2014; Choi et al. 2019; Sánchez‐Bayo and Wyckhuys 2021). On individual grassland species level, invertebrate decline affects abundance and flowering phenology of some species, causing shifts of peak flowering times (Ulrich et al. 2020). And at the microbiome level, the invertebrate decline reduces richness and β‐diversity and alters community composition of bacteria associated with leaves and flowers of grasslands (Junker et al. 2021). These results, as the Insect Armageddon experiment itself, were focused on the grassland communities. But, with the addition of clonal oaks, we expanded the analyses to effects of invertebrate decline on a (in this case) non‐target plant species with the highly‐standardised and well‐established phytometer system of the *Q. robur* DF159 clone (Herrmann, Munch, and Buscot 1998).

### 4.1 Invertebrate Decline Shifted the Leaf Microbiome Despite Low Display of Herbivory

Herbivory is one of the fundamental plant‐invertebrates‐microbiome interactions, however, the oak leaves did not show any significant signs of herbivory under any of the invertebrate biomass treatments at the time of sampling. While oaks have been described as attracting a vast diversity of invertebrates (Brändle and Brandl 2001), the invertebrates collected and transferred to the iDiv Ecotron originated from a meadow, hence, *Q. robur* was likely not a target species. Even the aphid infestation in response to reduced total invertebrate biomass, manifested itself primarily within the grassland communities. The lack of herbivory was confirmed by the gene expression analysis—response of herbivory related gene expression to invertebrate treatments was minimal. Gene selection was based on previous work (Bacht et al. 2019) on defence mechanisms in oaks induced by gypsy moth *Lymantria dispar*. In other settings, perhaps a broadened selection of genes should be considered, i.e. more universal genes such as for chitinase to detect herbivory related responses.

Herbivores have been shown to also exert control on microbial dynamics (Burkepile and Thurber 2019). Even though herbivory response was not strong, some herbivory likely took place and enabled input of microbiota to leaves through feeding. This is suggested by leave microbiomes showing higher susceptibility to invertebrate biomasses than roots and rhizosphere microbiomes. Bacterial communities on leaves had significantly higher OTU richness when invertebrates were present, but with the highest OTU richness at the 36% invertebrate biomass treatment, coinciding with the highest aphid biomasses. Also at 36% IB, abundance of *Bdellovibrionota* increased, which are predatory bacteria and are considered potential biocontrol agents preying on plant pathogens (Martins et al. 2022). As these increases in abundance were primarily at the 36% IB, we assume they were primarily a reaction to the aphid infestation as well.

Even though fungal richness did not respond to invertebrate biomass, we observed a significantly higher abundance of *Exobasidiomycetes* and *Ustilagomycetes* at 100% IB and for *Ustilagomycetes* even higher at 36% IB—again coinciding with reduced invertebrate but increased aphid biomass. *Exobasiodiomycetes* and *Ustilagomycetes* have been reported as plant pathogens, i.e. causing smut disease in plants (Bauer et al. 2001; Begerow, Lutz, and Oberwinkler 2002). But, these plant pathogens have not been reported as causing severe diseases in oaks, thus likely living as yeasts on the oak leaf after transmission from aphids.

Compared to Junker et al. (2021), who found decreasing bacterial richness for leaves of grassland species in the same experimental setup, the oak microbiome seems more stable against top‐down invertebrate biomass effects. Other studies also found the presence of insects to alter leaf microbiomes which further could be linked to shifts in the rhizosphere microbiome (Humphrey and Whiteman 2020; Das, Bhattacharyya, and Bhar 2023), which we could not clearly replicate with the oak clones. As the strongest shifts were found at 36% IB and highest aphid biomass, we suggest that not invertebrate biomass alone, but the concurrence of increased pest infestation caused by reduced top‐down controls determine the extent of implications of invertebrate declines on aboveground oak associated microbiomes. Yet, collected invertebrates came from grasslands and therefore should expectedly have a higher impact on grassland plant species than on oaks. Junker et al. (2021) further showed, bacteria to react rather to invertebrate biomass than invertebrate abundances (100%/36%/0%), highlighting the importance of the identity of present invertebrates. Further, shifts in plant‐associated microbiota are not caused solely by external factors like herbivory, but also indirect niche‐shaping factors like plants' responses to interactions (Junker and Keller 2015).

### 4.2 Belowground Fungi and Bacteria Are Unequally Affected by Invertebrate Abundance

Loss of invertebrate biomass could have direct or indirect consequences on ecosystem processes. The alpha diversities of fungal and bacterial belowground oak communities were not susceptible to reduced IB, suggesting any changes in microbial community compositions happened rather by internal changes then from input or loss of microbial species. Impacts of IB on microbial β‐diversity and taxonomic profiles were more pronounced in fungal than in bacterial communities, but either way not strong. Confirming previous studies, we found belowground fungal communities in oak roots and rhizosphere to be taxonomically similar, while leaf fungal communities showed differences in taxonomy as well as α‐ and β‐diversity. Root and rhizosphere fungal microbiomes were predominantly formed by ectomycorrhizal fungi, which was previously shown for the DF159 clones planted in grassland field sites, where the community composition was also highly affected by soil and weather parameters (Habiyaremye et al. 2020). The class *Dothideomycetes*, of which many taxa are saprotrophic, was slightly less abundant in roots at 100% IB treatment, while we would expect the opposite in response to higher availability of invertebrate carcasses. However, at 100% IB treatment, the chemoheterotrophic genus *Blastocella* was slightly less abundant which is in line with reports that these taxa are rather found in nutrient limited environments (Huber et al. 2017), here suggesting *Blastocella* responds to the reduced nutrient input from lower insect biomasses. Ectomycorrhizal fungi majorly formed belowground fungal communities, explaining the high similarity between roots and rhizosphere and showing successful inoculation of oak sapling with *P. croceum* before transplanting. With fungi being able to acquire nitrogen of which one source is invertebrate carcass, we expected to see shifts in mycorrhizal fungi. However, we did not find any significant shifts among fungal guilds in response to the invertebrate decline. Again, this suggests the most ecosystem implications caused by an invertebrate decline are indirect and caused by the reduced aboveground pest control and are more visible it the primary target grasslands.

In contrast to fungi, bacterial communities differed more strongly across all three oak compartments in α‐ and β‐diversity with leaves and rhizosphere differentiating the most from each other taxonomically. Roots and leaves were majorly inhabited by *Oxyphotobacteria* belonging to *Cyanobacteria*, which have often been found to promote plant growth through nitrogen fixation and production of bio‐control agents effective against soil‐borne fungal pathogens (Prasanna et al. 2013). However, we could not make a conclusion of bacteria responding either directly to invertebrate biomass or indirectly via changes in nutrient input. We assumed, bacterial soil communities might indirectly be affected by aphid infestation, but neither in our nor other studies (O'Brien et al. 2018), bacterial α‐ and β‐diversity responded to presence of aphids. However, we did see the abundance of bacteria candidate phylum FCPU426 to increase belowground at 100% IB. FCPU426 was shown to respond positively to *N* input and was correlated with plant defence mechanisms (Pineda et al. 2020; Sun et al. 2019), showing the level of invertebrate biomass does alter soils. We suspect, effects of invertebrate decline on soils and soil communities were subdued by the tubes the oak saplings were kept in throughout the experiment, limiting any exchange with the surrounding grassland community.

We expected to see shifts in the oak‐associated microbial communities in response to declining invertebrate biomass accompanied by changes in nutrient input. Eisenhauer et al. (2023) for the same experiment reported higher plant nitrogen and potassium with decreasing invertebrate abundance, but lower plant carbon at low invertebrate abundance for grassland species. Further, plant tissue carbon concentration and mean nitrogen concentration in through fall traps significantly decreased with decreasing invertebrate biomass (Eisenhauer et al. 2023). Some of these changes likely were introduced by aphids infestation, which can cause changes in nitrogen and phosphorus‐nitrogen ratio available for host plants with consequences for mycorrhizal‐aphid‐plant interactions (Wang et al. 2020). Nevertheless, none of these altered nutrient availabilities translated to the oak‐associated microbiome.

### 4.3 Impacts of Invertebrate Decline on Oak Performance

The clonal oak DF159 was chosen to ensure highest comparability between experimental units at the iDiv Ecotron. Even though growing conditions and genetic profile of the oak saplings were identical, the oaks varied strongly in performance across branch, roots and leaf growth. Overall, the oaks did not show significant variation in performance in response to invertebrate biomass, contrarily to the standardised grassland in the Ecotron (Ulrich et al. 2020; Eisenhauer et al. 2023; Junker et al. 2021). Contrary to grasslands, we could not show any shifts in biomass allocation in the oak saplings as a response to declining invertebrate biomass.

Even though our study did not show a strong response of *Q. robur* and its microbiome to an imminent invertebrate decline, the multitrophic implications of such declines need to be further studied. Several studies showed oaks and forests in general to be severely affected by climate extremes and defoliating invertebrates plus increases in pests (Hartmann, Blank, and Lewark 1989; Siwecki, Polska Akademia Nauk, and Liese 1991; Thomas, Blank, and Hartmann 2002; Thomas 2008). While our design with grassland ecosystems only showed minimal indirect effects of invertebrate decline on the non‐target oak saplings, such indirect effects could become more severe over a longer time period of reduced invertebrate biomass. Especially when invertebrate decline goes along with decreased pest control and in combination with climate change this could have cascading and devastating impacts on tree health and whole ecosystems (Pureswaran, Roques, and Battisti 2018). With the oak being one of the most ecologically and economically important trees in European forests, ongoing loss would cause huge damages ecologically and economically (Thomas 2008).

## 5 Conclusion

Here, we report evidence that the loss of invertebrate species and biomass has consequences on ecosystem processes and services. As a direct consequence, invertebrate loss reduces aboveground pest control. This exposes plants to more potential threats, here a severe aphids infestation at reduced invertebrate biomass. Indirectly, invertebrate loss shifts microbial communities in plants and soil, potentially affecting nutrient cycling and decomposition processes. Even though the here studied oaks were not the primary target plant, we see shifts in their associated microbiomes. Especially fungal communities respond to invertebrate loss with an accumulation of parasites and pathogens in relation to an increase in aboveground pests. Our findings establish a strong difference between above‐ and belowground, with the impacts of invertebrate decline being more pronounced in the leaf microbiome. Our study shows that invertebrate loss has—though minimal—far reaching impacts on ecosystem that reach secondary target plants and their microbiome.

## Author Contributions

**Cynthia Albracht:** investigation, data curation, formal analysis, writing – original draft, visualization. **François Buscot:** conceptualization, funding acquisition, writing – review and editing. **Nico Eisenhauer:** conceptualization, funding acquisition, writing – review and editing. **Alban Gebler:** conceptualization, data curation, project administration, investigation, writing – review and editing. **Sylvie Herrmann:** conceptualization, funding acquisition, investigation, writing – review and editing. **Anja Schmidt:** conceptualization, investigation, writing – review and editing. **Mika Tarkka:** conceptualization, funding acquisition, writing – review and editing. **Kezia Goldmann:** conceptualization, formal analysis, supervision, writing – original draft.

## Conflicts of Interest

The authors declare no conflicts of interest.

## Supporting information


**Data S1.** Supporting Information.

## Data Availability

The raw sequencing data are available at NCBI under the accession number PRJNA913823. The R‐Code is available on Github: https://github.com/cyn‐alb/OakMicrobiome_InsectArmageddon.
