# Supplementary material for: Invertebrate Decline Has Minimal Effects on Oak‐Associated Microbiomes
Source: Environ Microbiol. 2025 Feb 12;27(2):e70051. doi: 10.1111/1462-2920.70051 (PMC11822092; doi:10.1111/1462-2920.70051)
Supplement: Supplementary file 1 — Data S1. Supporting Information. [file EMI-27-e70051-s001.docx]

## Appendix: Supplementary Material

### Invertebrate decline has minimal effects on oak-associated microbiomes

#### Running title: Effects of invertebrate decline on oak microbiome

Cynthia Albracht^1,2^, François Buscot^2,3^, Nico Eisenhauer^3,4^, Alban Gebler^3^, Sylvie Herrmann^2,3^, Anja Schmidt^5^, Mika Tarkka^2,3^, and Kezia Goldmann^2^

*^1^ Institute for Biosafety in Plant Biotechnology, Julius Kühn-Institut, Quedlinburg, Germany*

*^2^ Department of Soil Ecology, Helmholtz Centre for Environmental Research – UFZ Halle, Halle, Germany*

*^3^ German Centre for Integrative Biodiversity Research (iDiv) Halle-Jena-Leipzig, Leipzig, Germany*

*^4^ Institute of Biology, Leipzig University, Leipzig, Germany*

*^5^ Department of Conservation & Social-Ecological Systems, Helmholtz Centre for Environmental Research – UFZ Halle, Halle, Germany*

**Table S1** Results of soil analyses of soil from 0-30 cm depth of each Ecounit. IB % - invertebrate biomass; soils with (H) or without (N) added humus soil. Soil texture: sL – sandy loam, lS – loamy sand, l’S – slighty loamy sand; n.a. - below measurable content.

| Ecounit | **IB %** | **Soil** | **Dry mass** | **NO3-N** | | **NH4-N** | | **Soil texture** | **pH** | **org substance** | **Total N** | **C/N-Ratio** |
| --- | --- | --- | --- | --- | --- | --- | --- | --- | --- | --- | --- | --- |
|  |  |  | **%** | **mg/100g** | **kg/ha** | **mg/100g** | **kg/ha** |  |  | **%** | **%** |  |
| EU01 | 0 | n | 86.9 | 0.715 | 37 | <0,00 | n.a. | sL | 7.4 | 2.2 | 0.09 | 14 |
| EU02 | 36 | n | 84.6 | 0.727 | 39 | 0.011 | 1 | sL | 7.4 | 2.3 | 0.11 | 12 |
| EU03 | 100 | n | 90.4 | 0.410 | 20 | 0.012 | n.a. | lS | 7.4 | 1.1 | 0.05 | 13 |
| EU04 | 100 | n | 86.0 | 0.575 | 30 | 0.013 | 1 | sL | 7.5 | 1.6 | 0.07 | 13 |
| EU05 | 36 | n | 92.5 | 0.247 | 12 | 0.030 | 1 | l'S | 7.5 | 0.5 | <0.04 (+) | n.a. |
| EU06 | 100 | n | 88.1 | 0.702 | 36 | <0,00 | n.a. | sL | 7.5 | 2.2 | 0.09 | 14 |
| EU07 | 36 | n | 93.5 | 0.274 | 13 | <0,00 | n.a. | l'S | 7.4 | 0.6 | 0.04 | 9 |
| EU08 | 0 | n | 94.3 | 0.228 | 11 | 0.013 | n.a. | lS | 7.5 | 0.8 | 0.04 | 12 |
| EU09 | 36 | n | 95.6 | 0.336 | 16 | 0.016 | 1 | lS | 7.5 | 0.8 | 0.04 | 12 |
| EU10 | 0 | n | 94.6 | 0.268 | 13 | <0,00 | n.a. | lS | 7.5 | 0.8 | 0.04 | 12 |
| EU11 | 100 | n | 91.1 | 0.248 | 12 | <0,00 | n.a. | l'S | 7.5 | 0.8 | <0.04 (+) | n.a. |
| EU12 | 0 | n | 87.0 | 0.562 | 29 | <0,00 | n.a. | sL | 7.4 | 1.9 | 0.09 | 12 |
| EU13 | 36 | n | 95.3 | 0.280 | 13 | <0,00 | n.a. | lS | 7.3 | 0.9 | 0.04 | 13 |
| EU14 | 100 | h | 93.4 | 0.287 | 14 | <0,00 | n.a. | lS | 7.5 | 0.7 | <0.04 (+) | n.a. |
| EU15 | 0 | h | 94.6 | 0.278 | 13 | 0.011 | n.a. | lS | 7.5 | 0.8 | 0.04 | 12 |
| EU16 | 36 | n | 91.7 | 0.426 | 21 | <0,00 | n.a. | lS | 7.4 | 1.4 | 0.07 | 12 |
| EU17 | 36 | h | 94.5 | 0.302 | 14 | 0.012 | n.a. | l'S | 7.6 | 0.8 | <0.04 (+) | n.a. |
| EU18 | 0 | h | 92.8 | 0.343 | 16 | <0,00 | n.a. | lS | 7.5 | 1.5 | 0.06 | 15 |
| EU19 | 100 | n | 88.1 | 0.229 | 12 | <0,00 | n.a. | lS | 7.5 | 0.9 | <0.04 (+) | n.a. |
| EU20 | 100 | n | 93.4 | 0.219 | 11 | <0,00 | n.a. | lS | 7.5 | 0.7 | <0.04 (+) | n.a. |
| EU21 | 100 | h | 87.5 | 0.516 | 27 | <0,00 | n.a. | sL | 7.5 | 1.8 | 0.08 | 13 |
| EU22 | 36 | h | 92.4 | 0.367 | 18 | <0,00 | n.a. | lS | 7.5 | 1.1 | 0.06 | 11 |
| EU23 | 0 | n | 95.5 | 0.284 | 13 | <0,00 | n.a. | sL | 7.6 | 0.9 | 0.04 | 13 |
| EU24 | 0 | n | 94.2 | 0.258 | 12 | <0,00 | n.a. | lS | 7.6 | 0.8 | <0.04 (+) | n.a. |

***Table S2*** *Sequences of primers used in qRT-PCR for herbivory related genes in Quercus robur L. as described by Bacht et al. (2019)*

| **Primer Name** | **Primer Seq.** | **Gene ID** | **Gene description** |
| --- | --- | --- | --- |
| **Qr_13LOX3_F3** | CAGGGAATGGCTGTTGAGGA | Qrob_P0338490.2 | 13-lipoxygenase |
| **Qr_13LOX3_R3** | CGGATACCACCCACACGATT |  |  |
| **Qr_AOC_F7** | AAGACCCACCAACGTTCAGG | Qrob_P0758490.2 | allene oxide cyclase |
| **Qr_AOC_R7** | TCCCCAACCGTTTCTCCAAG |  |  |
| **Qr_Far4_F21** | ATGCAATGGACTCTGTGGCA | Qrob_P0119250.2 | alpha farnesene synthase |
| **Qr_Far4_R21** | GTGAGGCCTCCAAAAGCTCA |  |  |
| **Qr_HPL4_F27** | CTGAGGAGTTTGTGGGCCAT | Qrob_P0010750.2 | hydroperoxide lyase |
| **Qr_HPL4_R27** | TTCGCCGGACACTGCTTATT |  |  |
| **Qr_CHI_F37** | GTGTCAGCAGCAGAAGGAGT | comp42425_c1_ seq11 | chitinase I |
| **Qr_CHI_R37** | CCACAGACCCCACCATTTCA |  |  |
| **18S_2-f** | CAAGGTGGACTCTCTCACGG |  | 18S rRNA |
| **18S_2-r** | CCTCGGATGCAGAACACC |  |  |

***Table S3*** *Overview of performance of individual oak saplings: stage – growth stage of plant; plant stage –developmental stage of apical bud:* *A_rest=1; B_swelling=2; C_elongating=3; D_expansion=4; BD_dormand=5 ; branch stage - A_rest; B_swelling; C_stem elongation; D_leaf expansion; DB_dormand; PR_FW – fresh weight of primary root (g); LR_FW – fresh weight of lateral roots (g); LR_myco – mycorrhization rate of lateral roots (%); BR_length – total length of all branches (cm); LV_FW – fresh weight of leaves (g)*

| *IB* | *Oak_ID* | *Plant stage* | *Branch stage* | *PR_FW* | *LR_FW* | *LR_myco* | *BR_length* | *LV_FW* |
| --- | --- | --- | --- | --- | --- | --- | --- | --- |
| 0% | *ECO_18_1* | DB | B | 76.55 | 19.17 | 69.98 | 77.50 | 6.44 |
|  | *ECO_18_10* | 3A | n.a. | 24.78 | 1.73 | 29.35 | 63.00 | 5.53 |
|  | *ECO_18_12* | 2A | A | 25.04 | 13.91 | 31.33 | 15.86 | 3.15 |
|  | *ECO_18_15* | 2D | D | 25.64 | 6.48 | 63.10 | 43.40 | 4.17 |
|  | *ECO_18_18* | n.a. | B+D | 44.54 | 9.73 | 67.21 | 20.13 | 4.34 |
|  | *ECO_18_23* | 2D | A+B+D | 27.56 | 5.6 | 72.13 | 46.83 | 2.80 |
|  | *ECO_18_24* | DB | C | 18.23 | 6.52 | 60.51 | 57.00 | 4.20 |
|  | *ECO_18_8* | 2A | B+C | 36.26 | 26.1 | 18.87 | 16.79 | 3.92 |
| 36% | *ECO_18_13* | 2B | B | 21.72 | 10.69 | 46.16 | 19.00 | 4.01 |
|  | *ECO_18_16* | 3D | A | 40.18 | 5.02 | 53.09 | 38.63 | 6.76 |
|  | *ECO_18_17* | 2A | A+D | 18.86 | 9.29 | 56.98 | 44.00 | 4.38 |
|  | *ECO_18_2* | DB | D | 26.07 | 16.39 | 45.82 | 30.00 | 5.69 |
|  | *ECO_18_22* | DB | D | 30.84 | 9.26 | 37.47 | 14.08 | 3.94 |
|  | *ECO_18_5* | DB | D | 25.25 | 12 | 47.13 | 24.25 | 3.58 |
|  | *ECO_18_7* | 2D | A | 27.86 | 15.99 | 65.68 | 39.20 | 3.49 |
|  | *ECO_18_9* | 1C | A | 21.57 | 9.75 | 29.63 | 71.50 | 5.10 |
| 100% | *ECO_18_11* | DB | A | 26.92 | 3.79 | 44.44 | 12.42 | 2.03 |
|  | *ECO_18_14* | 1B | D | 24.72 | 18.69 | 38.38 | 46.33 | 4.85 |
|  | *ECO_18_19* | DB | C | 23.03 | 5.1 | 56.12 | 28.33 | 3.17 |
|  | *ECO_18_20* | DB | B | 25.77 | 13.92 | 82.48 | 9.50 | 4.64 |
|  | *ECO_18_21* | 2A | A+B | 40.27 | 17.04 | 55.64 | 53.50 | 3.19 |
|  | *ECO_18_3* | DB | D | 29.37 | 27.22 | 55.38 | 32.38 | 5.05 |
|  | *ECO_18_4* | DB | n.a. | 19.59 | 8.98 | 41.61 | 0.50 | 0.35 |
|  | *ECO_18_6* | 2D | D | 25.77 | 10.07 | 50.00 | 42.38 | 3.39 |

***Table S4*** *Tests for effects of invertebrate and aphids biomass on soil pH and organic substances in the EcoUnits and effects of total invertebrate and aphids biomass on alpha diversity of fungi and bacteria.*

|  | **pH** | | | **organic substances** | | |
| --- | --- | --- | --- | --- | --- | --- |
|  | **Df** | **F** | **P** | **Df** | **F** | **P** |
| **aphids biomass** | 1 | 0.03 | 0.85 | 1 | 0.15 | 0.69 |
| **total invertebrate biomass** | 1 | 0.09 | 0.76 | 1 | < 0.01 | 0.99 |
|  | **Fungi** | | | **Bacteria** | | |
|  | **Df** | **F** | **P** | **Df** | **F** | **p** |
| **aphids biomass** | 1 | 1.87 | 0.18 | 1 | 0.28 | 0.60 |
| **iinvertebrate biomass** | 1 | 2.75 | 0.10 | 1 | 0.27 | 0.61 |

***Table S5*** *Results of linear models for alpha diversity indices of (A) fungi and (B) bacteria in oak compartments against invertebrate biomass. Significant effects (p < 0.5) are bold.*

| Samples | Response variable | Df | F value | P values |
| --- | --- | --- | --- | --- |
| A Fungi |  |  |  |  |
| - Leaves | OTU richness | 2 | 0.68 | 0.52 |
|  | Shannon | 2 | 0.53 | 0.59 |
|  | Evenness | 2 | 0.33 | 0.72 |
| - Roots | OTU richness | 2 | 1.01 | 0.38 |
|  | Shannon | 2 | 0.37 | 0.69 |
|  | Evenness | 2 | 0.30 | 0.74 |
| - Rhizosphere | OTU richness | 2 | 0.62 | 0.55 |
|  | Shannon | 2 | 0.86 | 0.44 |
|  | Evenness | 2 | 0.91 | 0.42 |
| B Bacteria |  | 2 |  |  |
| - Leaves | OTU richness | 2 | 1.85 | 0.19 |
|  | Shannon | 2 | 2.89 | *0.08* |
|  | Evenness | 2 | 2.77 | *0.09* |
| - Roots | OTU richness | 2 | 0.49 | 0.62 |
|  | Shannon | 2 | 0.74 | 0.49 |
|  | Evenness | 2 | 0.91 | 0.42 |
| - Rhizosphere | OTU richness | 2 | 0.37 | 0.69 |
|  | Shannon | 2 | 0.26 | 0.77 |
|  | Evenness | 2 | 0.16 | 0.85 |

***Table S6*** ***Differential abundances of microbial classes and functional guilds in response to invertebrate biomass levels.*** *(A) Differential abundances fungi at class level; (B) differential abundances of bacteria at phylum level. Differential abundances were calculated as differences at 36% and 100% IB in comparison to control with 0% IB individually for oak leaves, roots and rhizosphere. Only significant differences are given as fold change values, fungal classes which were not present in the respective compartment are listed as empty cells.*

|  | **Leaves** | | **Roots** | | **Rhizosphere** | |
| --- | --- | --- | --- | --- | --- | --- |
|  | **36%** | **100%** | **36%** | **100%** | **36%** | **100%** |
| **A Fungi** |  |  |  |  |  |  |
| *Agaricomycetes* | 0.00 | 0.00 | 0.00 | 0.00 | 0.00 | 0.00 |
| *Agaricostilbomycetes* | 0.00 | 0.00 |  |  | 0.00 | 0.00 |
| *Aphelidiomycetes* |  |  |  |  | 0.00 | 0.00 |
| *Archaeosporomycetes* |  |  |  |  | 0.00 | 0.00 |
| *Atractiellomycetes* |  |  |  |  | 0.00 | 0.00 |
| *Basidiobolomycetes* |  |  | 0.00 | 0.00 | 0.00 | 0.00 |
| *Classiculomycetes* |  |  |  |  | 0.00 | 0.00 |
| *Cladochytriomycetes* |  |  | 0.00 | 0.00 |  |  |
| *Cystobasidiomycetes* | 0.00 | 0.00 | 0.00 | 0.00 | 0.00 | 0.00 |
| *Dothideomycetes* | 0.00 | 0.00 | 0.00 | **-1.19** | 0.00 | 0.00 |
| *Endogonomycetes* |  |  |  |  | 0.00 | 0.00 |
| *Entomophthoromycetes* | 0.00 | 0.00 |  |  | 0.00 | 0.00 |
| *Eurotiomycetes* | 0.00 | 0.00 | 0.00 | 0.00 | 0.00 | 0.00 |
| *Exobasidiomycetes* | **2.99** | **3.25** |  |  |  |  |
| *Geminibasidiomycetes* |  |  |  |  | 0.00 | 0.00 |
| *Glomeromycetes* | 0.00 | 0.00 | 0.00 | 0.00 | 0.00 | 0.00 |
| *Laboulbeniomycetes* | 0.00 | 0.00 | 0.00 | 0.00 | 0.00 | 0.00 |
| *Lecanoromycetes* | 0.00 | 0.00 | 0.00 | 0.00 | 0.00 | 0.00 |
| *Leotiomycetes* | 0.00 | 0.00 | 0.00 | 0.00 | 0.00 | 0.00 |
| *Malasseziomycetes* | 0.00 | 0.00 |  |  |  |  |
| *Microbotryomycetes* | **2.16** | 0.00 | 0.00 | 0.00 | 0.00 | 0.00 |
| *Mortierellomycetes* | 0.00 | 0.00 | 0.00 | 0.00 | 0.00 | 0.00 |
| *Mucoromycetes* |  |  |  |  | 0.00 | 0.00 |
| *Orbiliomycetes* | 0.00 | 0.00 | 0.00 | 0.00 | 0.00 | 0.00 |
| *Paraglomeromycetes* |  |  |  |  | 0.00 | 0.00 |
| *Pezizomycetes* | 0.00 | 0.00 | 0.00 | 0.00 | 0.00 | 0.00 |
| *Pucciniomycetes* |  |  |  |  | 0.00 | 0.00 |
| *Rhizophlyctidomycetes* |  |  |  |  | 0.00 | 0.00 |
| *Rhizophydiomycetes* |  |  |  |  | 0.00 | 0.00 |
| *Saccharomycetes* | 0.00 | 0.00 | 0.00 | 0.00 | 0.00 | 0.00 |
| *Sordariomycetes* | 0.00 | 0.00 | 0.00 | 0.00 | 0.00 | 0.00 |
| *Spizellomycetes* | 0.00 | 0.00 | 0.00 | 0.00 | 0.00 | 0.00 |
| *Tremellomycetes* | 0.00 | 0.00 | 0.00 | 0.00 | 0.00 | 0.00 |
| *Umbelopsidomycetes* |  |  |  |  | 0.00 | 0.00 |
| *Ustilaginomycetes* | **4.12** | **3.61** |  |  |  |  |
| *Zoopagomycetes* |  |  |  |  | 0.00 | 0.00 |
|  | **Leaves** | | **Roots** | | **Rhizosphere** | |
|  | **36%** | **100%** | **36%** | **100%** | **36%** | **100%** |
| **B Bacteria** |  |  |  |  |  |  |
| *Abditibacteriota* | 0.00 | 0.00 | 0.00 | 0.00 | 0.00 | 0.00 |
| *Acidobacteriota* | 0.00 | 0.00 | 0.00 | 0.00 | 0.00 | **- 0.39** |
| *Actinobacteriota* | 0.00 | 0.00 | 0.00 | 0.00 | 0.00 | 0.00 |
| *Armatimonadota* | 0.00 | 0.00 | 0.00 | 0.00 | 0.00 | 0.00 |
| *Bacteroidota* | 0.00 | 0.00 | 0.00 | 0.00 | 0.00 | 0.00 |
| *Bdellovibrionota* | **1.53** | 0.00 | 0.00 | 0.00 | 0.00 | 0.00 |
| *Chloroflexi* | 0.00 | 0.00 | 0.00 | 0.00 | 0.00 | 0.00 |
| *Cyanobacteria* | 0.00 | 0.00 | 0.00 | 0.00 | 0.00 | 0.00 |
| *Deinococcota* | 0.00 | 0.00 | 0.00 | 0.00 | 0.00 | 0.00 |
| *Dependentiae* |  |  | 0.00 | 0.00 | 0.00 | 0.00 |
| *Desulfobacterota* |  |  | 0.00 | 0.00 | 0.00 | 0.00 |
| *Elusimicrobiota* |  |  | 0.00 | 0.00 | 0.00 | 0.00 |
| *Entotheonellaeota* |  |  |  |  | 0.00 | 0.00 |
| *FCPU426* |  |  | 0.00 | **1.54** | 0.00 | **1.47** |
| *Fibrobacterota* |  |  | 0.00 | 0.00 | 0.00 | 0.00 |
| *Firmicutes* | 0.00 | 0.00 | 0.00 | 0.00 | 0.00 | 0.00 |
| *Fusobacteriota* |  |  | 0.00 | 0.00 |  |  |
| *GAL15* |  |  |  |  | 0.00 | 0.00 |
| *Gemmatimonadota* |  |  | 0.00 | 0.00 | 0.00 | 0.00 |
| *Halanaerobiaeota* |  |  |  |  | 0.00 | 0.00 |
| *Hydrogenedentes* |  |  | 0.00 | 0.00 | 0.00 | 0.00 |
| *Latescibacterota* |  |  | 0.00 | 0.00 | 0.00 | 0.00 |
| *Margulisbacteria* |  |  | 0.00 | 0.00 | 0.00 | 1.58 |
| *MBNT15* |  |  | 0.00 | 0.00 | 0.00 | 0.00 |
| *Methylomirabilota* |  |  | 0.00 | 0.00 | 0.00 | 0.00 |
| *Myxococcota* | 0.00 | 0.00 | 0.00 | 0.00 | 0.00 | 0.00 |
| *NB1-j* |  |  | 0.00 | 0.00 | 0.00 | 0.00 |
| *Nitrospirota* |  |  | 0.00 | 0.00 | 0.00 | 0.00 |
| *Patescibacteria* | 0.00 | 0.00 | 0.00 | 0.00 | 0.00 | 0.00 |
| *Planctomycetota* | 0.00 | 0.00 | 0.00 | 0.00 | 0.00 | 0.00 |
| *Proteobacteria* | 0.00 | 0.00 | 0.00 | 0.00 | 0.00 | 0.00 |
| *RCP2-54* |  |  | 0.00 | 0.00 | 0.00 | 0.00 |
| *SAR324_clade(Marine_group_B)* |  |  | 0.00 | 0.00 | 0.00 | 0.00 |
| *Spirochaetota* |  |  | 0.00 | 0.00 | 0.00 | 0.00 |
| *Sumerlaeota* |  |  | 0.00 | 0.00 | 0.00 | 0.00 |
| *Verrucomicrobiota* | 0.00 | 0.00 | 0.00 | 0.00 | 0.00 | 0.00 |
| *WPS-2* | 0.00 | 0.00 | **- 1.31** | 0.00 | 0.00 | 0.00 |
| *WS2* |  |  | 0 | 0 | 0 | 0 |
|  | **Leaves** | | **Roots** | | **Rhizosphere** | |
|  | **36%** | **100%** | **36%** | **100%** | **36%** | **100%** |
| **C Fungal Guilds** |  |  |  |  |  |  |
| ectomycorrhizal | 0.00 | 0.00 | 0.00 | 0.00 | 0.00 | 0.00 |
| mycoparasite | 0.00 | 0.00 | 0.00 | 0.00 | 0.00 | 0.00 |
| sooty mold | 0.00 | 0.00 | 0.00 | 0.00 | 0.00 | 0.00 |
| unspecified saprotroph | 0.00 | 0.00 | 0.00 | 0.00 | 0.00 | 0.00 |
| litter saprotroph | 0.00 | 0.00 | 0.00 | 0.00 | 0.00 | 0.00 |
| animal parasite | 0.00 | 0.00 | 0.00 | 0.00 | 0.00 | 0.00 |
| wood saprotroph | 0.00 | 0.00 | 0.00 | 0.00 | 0.00 | 0.00 |
| soil saprotroph | 0.00 | 0.00 | 0.00 | 0.00 | 0.00 | 0.00 |
| plant pathogen | 0.00 | 0.00 | 0.00 | 0.00 | 0.00 | 0.00 |
| epiphyte | 0.00 | 0.00 | 0.00 | 0.00 | 0.00 | 0.00 |
| dung saprotroph | 0.00 | 0.00 | 0.00 | 0.00 | 0.00 | 0.00 |
| foliar endophyte | 0.00 | 0.00 | 0.00 | 0.00 | 0.00 | 0.00 |
| root endophyte | 0.00 | 0.00 | 0.00 | 0.00 | 0.00 | 0.00 |
| nectar/tap saprotroph | 0.00 | 0.00 | 0.00 | 0.00 | 0.00 | 0.00 |
| arbuscular mycorrhizal | 0.00 | 0.00 | 0.00 | 0.00 | 0.00 | 0.00 |
| pollen saprotroph | 0.00 | 0.00 | 0.00 | 0.00 | 0.00 | 0.00 |
| unspecified | 0.00 | 0.00 | 0.00 | 0.00 | 0.00 | 0.00 |
| lichen parasite | 0.00 | 0.00 | 0.00 | 0.00 | 0.00 | 0.00 |
| lichenized | 0.00 | 0.00 | 0.00 | 0.00 | 0.00 | 0.00 |

**Table S7** Biomass [g], abundance, and richness of invertebrates [per sample] added to the EcoUnits during the four invertebrate addition campaigns for each invertebrate treatment (36 vs. 100 [%]); adapted from Eisenhauer et al 2023, Current Biology. Note that these samples were only used to determine the biomass, abundances, and richness of invertebrates, but were not added to EcoUnits to avoid any potential negative effects on their health. SE = Standard error. Three samples were extrapolated before calculating invertebrate abundance, biomass and richness, to guarantee consistent sampling time. To account for different sample collection methods, we created pairs of available Malaise samplings and 1 cup catch per Malaise for each treatment-sampling round combination creating 100 randomized datasets of pairings. For further details on methods, please refer to Eisenhauer et al 2023, Current Biology. From these randomized dataset, we calculated mean and SE, resulting in 100 mean values of richness, abundance and biomass.

| *Invertebrate biomass* | | | | | | |
| --- | --- | --- | --- | --- | --- | --- |
| **sampling round** | **mean biomass 36%** | **SE biomass 36%** | **mean biomass 100%** | **SE biomass 100%** | **% biomass in 36%** | **% reduction in 36%** |
| **May** | 0,95 | 0,09 | 2,07 | 0,82 | 46 | -54 |
| **June** | 0,88 | 0,35 | 3,96 | 1,07 | 22 | -78 |
| **July** | 0,56 | 0,29 | 1,35 | 0,77 | 41 | -59 |
| **September** | 0,57 | 0,42 | 1,61 | 0,34 | 35 | -65 |
|  |  |  |  |  | **36** | **-64** |
| *Invertebrate abundance* | | | | | | |
| **sampling round** | **mean abund. 36%** | **SE abund. 36%** | **mean abund. 100%** | **SE abund. 100%** | **% abund. in 36%** | **% reduction in 36%** |
| **May** | 96,33 | 21,59 | 282,00 | 24,00 | 34 | -66 |
| **June** | 93,95 | 43,86 | 319,48 | 132,52 | 29 | -71 |
| **July** | 46,78 | 35,05 | 155,59 | 86,63 | 30 | -70 |
| **September** | 28,38 | 7,86 | 103,80 | 35,59 | 28 | -73 |
|  |  |  |  |  | **30** | **-70** |
| *Invertebrate richness* | | | | | | |
| **sampling round** | **mean richness 36%** | **SE richness 36%** | **mean richness 100%** | **SE richness 100%** | **% richness in 36%** | **% reduction in 36%** |
| **May** | 33,44 | 4,88 | 47,50 | 1,50 | 70 | -30 |
| **June** | 23,87 | 2,30 | 64,46 | 8,69 | 37 | -63 |
| **July** | 22,87 | 13,63 | 42,34 | 20,44 | 54 | -46 |
| **September** | 10,13 | 0,71 | 16,12 | 5,44 | 63 | -37 |
|  |  |  |  |  | **56** | **-44** |

**Table S8** Mean invertebrate abundances and richness of the aboveground invertebrate communities in the EcoUnits added in May 2018. Split into gross taxonomic groups for the 36 % and 100 % invertebrate treatments. Adapted from Eisenhauer et al 2023, Current Biology.

| **Taxonomic groups** | **Abundance 36%** | **Abundance 100%** | **Richness 36%** | **Richness 100%** |
| --- | --- | --- | --- | --- |
| *Aphidina* | 6 | 2 | 1 | 1 |
| *Araneae* | 1 | 5 | 1 | 5 |
| *Brachycera* | 126 | 144 | 13 | 4 |
| *Cicadina* | 15 | 19 | 4 | 8 |
| *Coleoptera* | 5 | 9 | 5 | 8 |
| *Formicidae* | 6 | 3 | 2 | 1 |
| *Gastropoda* | 5 | 11 | 1 | 1 |
| *Heteroptera* | 6 | 6 | 2 | 2 |
| *Sternorrhyncha* | 4 | 0 | 1 | 0 |
| *Hymenoptera* | 34 | 32 | 18 | 20 |
| *Lepidoptera* | 10 | 8 | 7 | 7 |
| *Nematocera* | 48 | 40 | 7 | 2 |
| *Neuroptera* | 1 | 1 | 1 | 1 |
| *Orthoptera* | 0 | 1 | 0 | 1 |
| **Total** | **276** | **281** | **63** | **61** |

**Table S9** Mean invertebrate abundances and richness of the aboveground invertebrate communities in the EcoUnits added in June 2018. Split into gross taxonomic groups for the 36 % and 100 % invertebrate treatments. Adapted from Eisenhauer et al 2023, Current Biology.

| **Taxonomic groups** | **Abundance 36%** | **Abundance 100%** | **Richness 36%** | **Richness 100%** |
| --- | --- | --- | --- | --- |
| *Aphidina* | 172 | 263 | 1 | 1 |
| *Araneae* | 5 | 35 | 4 | 10 |
| *Brachycera* | 48 | 199 | 11 | 20 |
| *Cicadina* | 8 | 54 | 7 | 16 |
| *Coleoptera* | 1 | 21 | 1 | 14 |
| *Collembola* | 0 | 4 | 0 | 1 |
| *Dermaptera* | 18 | 6 | 1 | 1 |
| *Formicidae* | 61 | 249 | 2 | 4 |
| *Gastropoda* | 3 | 51 | 1 | 1 |
| *Heteroptera* | 6 | 23 | 4 | 7 |
| *Hymenoptera* | 21 | 57 | 13 | 23 |
| *Isopoda* | 0 | 3 | 0 | 1 |
| *larvae* | 0 | 5 | 0 | 1 |
| *Lepidoptera* | 7 | 31 | 4 | 8 |
| *Nematocera* | 24 | 55 | 3 | 6 |
| *Orthoptera* | 0 | 1 | 0 | 1 |
| *Thysanoptera* | 13 | 13 | 2 | 3 |
| **Total** | **387** | **1070** | **54** | **118** |

**Table S10** Mean invertebrate abundances and richness of the aboveground invertebrate communities in the EcoUnits added in July 2018. Split into gross taxonomic groups for the 36 % and 100 % invertebrate treatments. Adapted from Eisenhauer et al 2023, Current Biology.

| **Taxonomic groups** | **Abundance 36%** | **Abundance 100%** | **Richness 36%** | **Richness 100%** |
| --- | --- | --- | --- | --- |
| *Aphidina* | 5 | 53 | 1 | 100% |
| *Araneae* | 5 | 72 | 5 | 8 |
| *Brachycera* | 66 | 151 | 13 | 16 |
| *Cicadina* | 8 | 56 | 6 | 13 |
| *Coleoptera* | 8 | 18 | 7 | 11 |
| *Collembola* | 0 | 2 | 0 | 1 |
| *Dermaptera* | 1 | 0 | 1 | 0 |
| *Diptera* | 1 | 0 | 1 | 0 |
| *Formicidae* | 10 | 100 | 2 | 4 |
| *Gastropoda* | 4 | 9 | 1 | 1 |
| *Heteroptera* | 1 | 9 | 1 | 6 |
| *Hymenoptera* | 36 | 92 | 20 | 36 |
| *Larvae* | 0 | 3 | 0 | 1 |
| *Lepidoptera* | 7 | 12 | 6 | 7 |
| *Nematocera* | 2 | 7 | 2 | 2 |
| *Neuroptera* | 0 | 1 | 0 | 1 |
| *Orthoptera* | 0 | 1 | 0 | 1 |
| **Total** | **154** | **586** | **66** | **109** |

**Table S11** Mean invertebrate abundances and richness of the aboveground invertebrate communities in the EcoUnits added in September 2018. Split into gross taxonomic groups for the 36 % and 100 % invertebrate treatments. Adapted from Eisenhauer et al 2023, Current Biology.

| **Taxonomic groups** | **Abundance 36%** | **Abundance 100%** | **Richness 36%** | **Richness 100%** |
| --- | --- | --- | --- | --- |
| *Aphidina* | 7 | 5 | 1 | 1 |
| *Araneae* | 6 | 10 | 3 | 3 |
| *Brachycera* | 32 | 221 | 8 | 10 |
| *Cicadina* | 0 | 3 | 0 | 3 |
| *Coleoptera* | 4 | 1 | 3 | 1 |
| *Formicidae* | 45 | 10 | 2 | 2 |
| *Gastropoda* | 1 | 0 | 1 | 0 |
| *Sternorrhyncha* | 0 | 13 | 0 | 1 |
| *Heteroptera* | 3 | 2 | 2 | 1 |
| *Hymenoptera* | 1 | 17 | 1 | 11 |
| *Larvae* | 1 | 1 | 1 | 1 |
| *Lepidoptera* | 2 | 17 | 2 | 4 |
| *Nematocera* | 1 | 21 | 1 | 2 |
| **Total** | **103** | **321** | **25** | **40** |

| **A**  **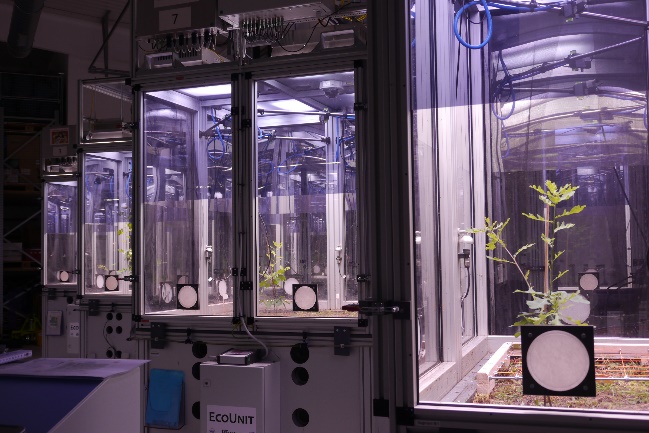** | **B**  **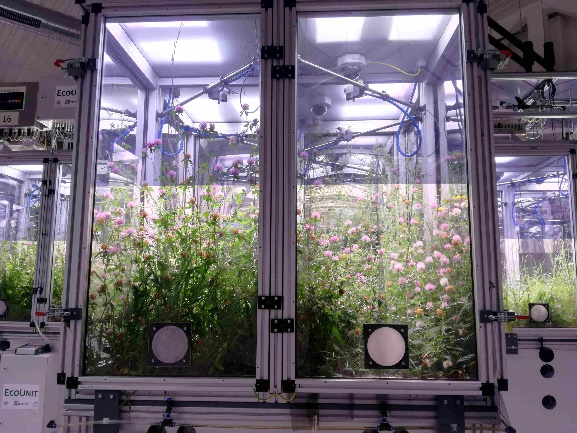** |
| --- | --- |
| **c**  **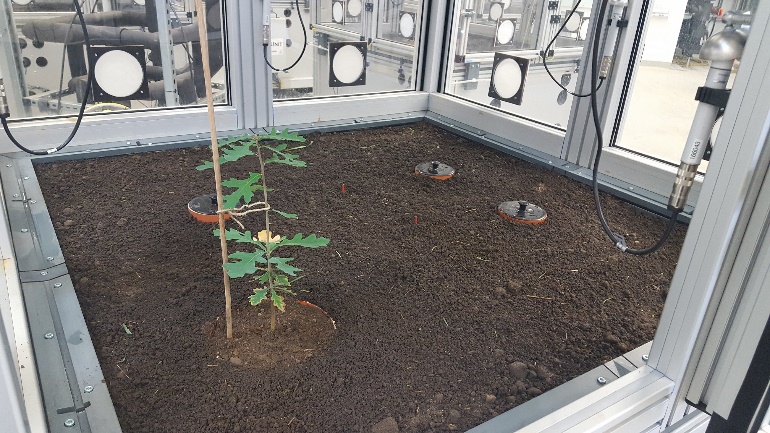** | **d**  **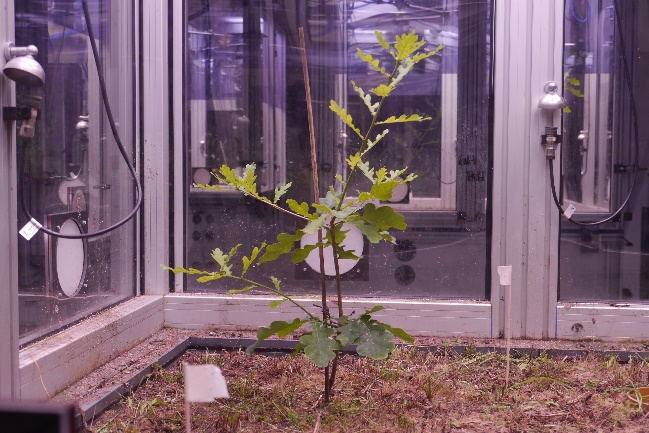** |

**Figure S1 Photos of the experimental setup.** a) overview of EcoUnits of the iDiv Ecotron; b) growth of artificial grassland community mid-experiment; c) oak saplings at time of planting, transferred to the EcoUnit inside plastic tube; d) oak sapling inside EcoUnit at time of harvest, when grassland was already harvested.

***
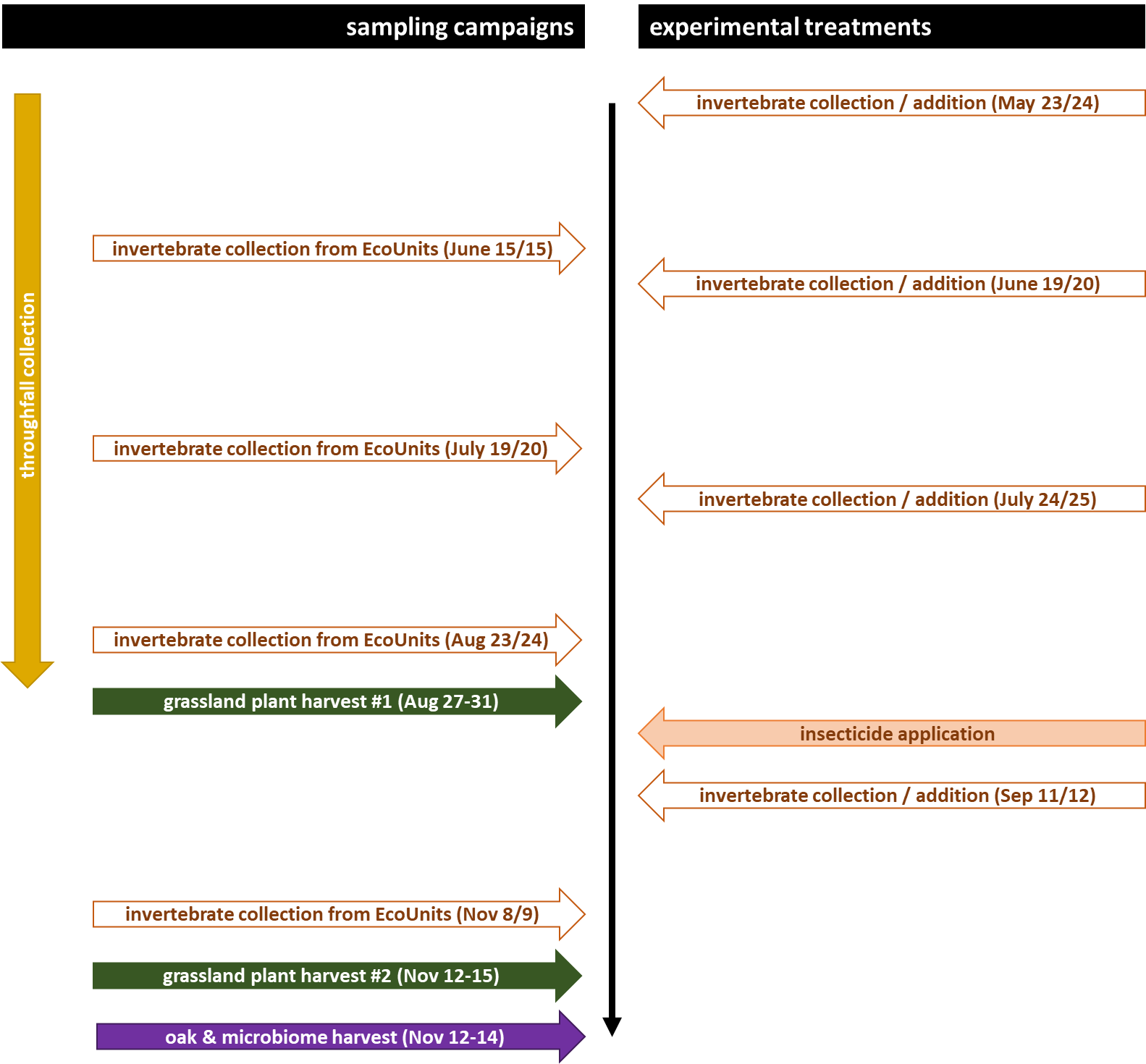
***

***Figure S2*** ***Schematic overview of the timeline of different samplings and experimental treatments including the seasonal exchange of invertebrate communities.*** *Left: sampling campaigns throughout the experiment; right: experimental treatments. Adapted from Eisenhauer et al 2023, Current Biology.*


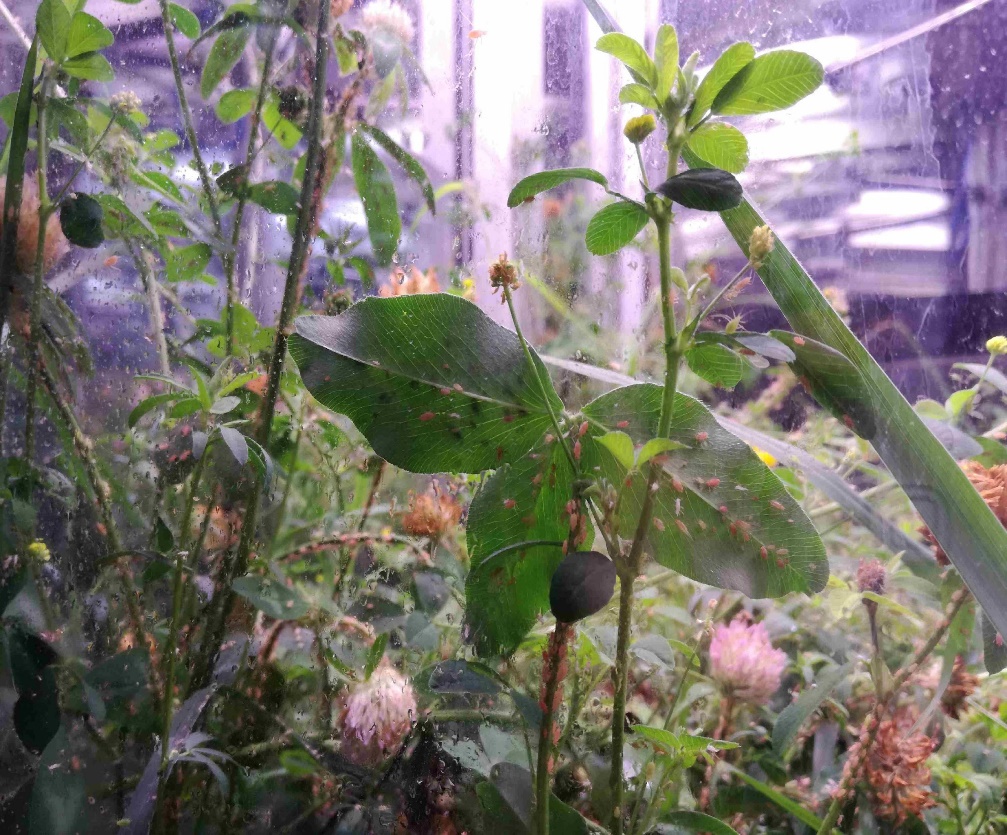

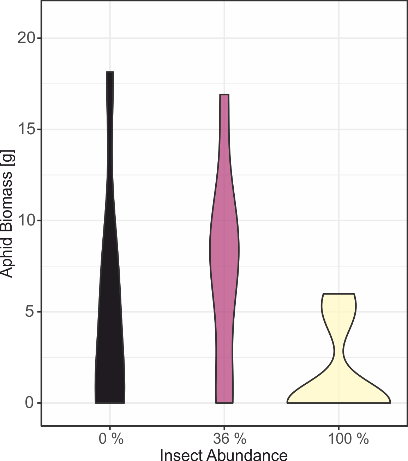

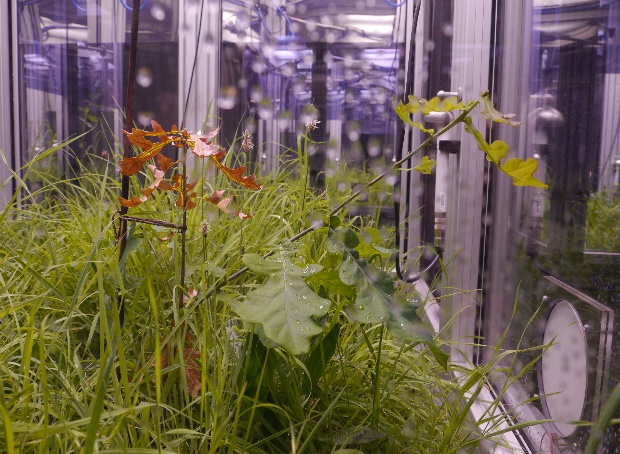


**A**

**B**

**C**

***Figure S3*** ***Depiction of aphid infestation within the EcoUnits in week 5-18 of the experiment.*** *(A) Photo of aphid infestation on grassland community in EcoUnit. (B) Aphids biomass [g] in EcoUnits per treatment showing increased infestation at low invertebrate levels. (C) Photo of EcoUnit at time of harvest in November with no more aphid infestation.*


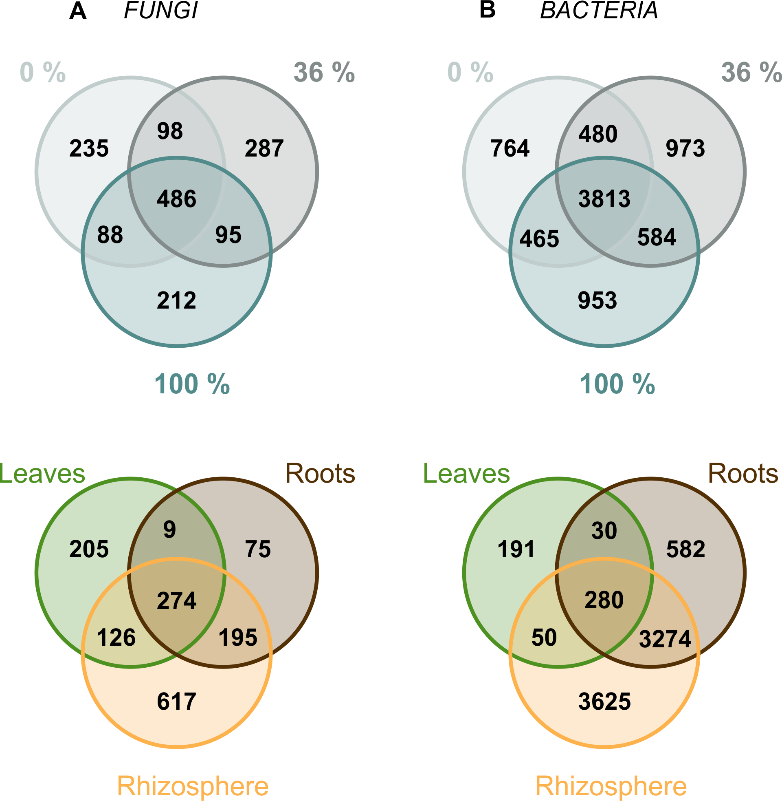


***Figure S4*** ***Venn diagrams of amount of shared and exclusive (A) fungal and (B) bacterial OTUs.*** *Top:* *OTUs present at all or individual invertebrate treatment levels (0% / 36% / 100%); bottom: OTUs present in all or individual oak compartments (leaves, roots and rhizosphere).*

*
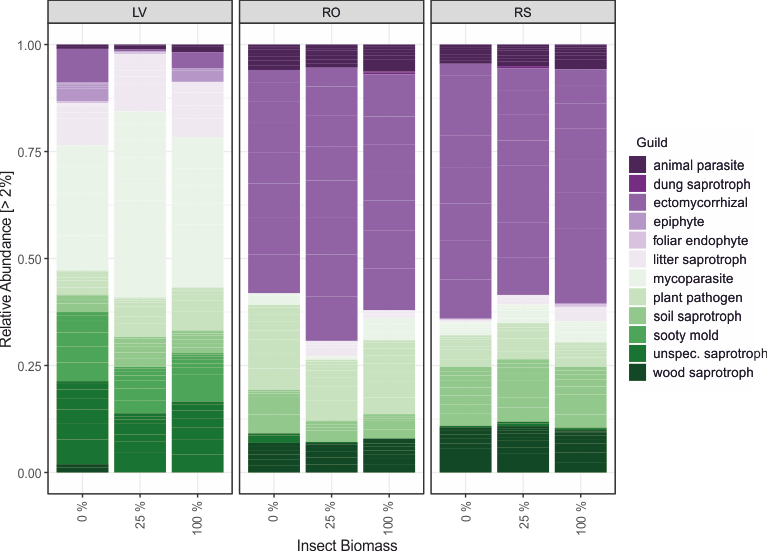
*

***Figure S5*** *Relative abundance of > 2% abundant fungal guilds in oak leaves (LV), roots (RO) and rhizosphere (RS) at different invertebrate abundances (0% / 36% / 100%).*
